# Preparation and Properties of Magnetically Responsive Graphene/Boron Nitride/Iron Oxide Filler Composite Epoxy Resin Materials

**DOI:** 10.3390/nano15120936

**Published:** 2025-06-16

**Authors:** Yiheng Yu, Duo Zhang, Hui He, Chaogui Luo, Ming Zhou

**Affiliations:** 1 School of Mechanical and Automotive Engineering, Guangxi University of Science and Technology, Liuzhou 545006, China; 18705590750@163.com (Y.Y.); 17755942221@163.com (D.Z.); 2 Guangxi TsingLube New Material Technology Co., Ltd., Liuzhou 545006, China; 17776690698@163.com (H.H.);

**Keywords:** thermal conductivity, magnetic field arrangement, thermal stability, ablation resistance

## Abstract

In this paper, magnetically responsive graphene/boron nitride/iron oxide fillers were prepared by growing iron oxide on the surface of graphene/boron nitride fillers via liquid-phase reaction. By adding the composite filler into the epoxy resin and utilising magnetic field-assisted curing, the composites were prepared to effectively improve the thermal conductivity of the composites while maintaining the insulating properties. The thermal conductivity of the composite filler is 2.1 Wm^−1^K^−1^, and the volume resistance is 4.63 × 10^12^ Ω·cm when the mass ratio of the composite filler is 25%, and the thermal stability and ablation resistance of the composites are improved compared with that of the pure epoxy resin.

## 1. Introduction

The development of high-power electronic devices [1], new energy equipment, and lightweight, protective encapsulation materials put forward higher requirements in thermal conductivity, thermal stability, and anti-corrosion performance [2].

Epoxy resin has been widely used in electronic packaging, structural bonding, composite matrices, and coatings due to its excellent mechanical properties [3], electrical insulation, adhesive properties, and good processing adaptability [4,5]. However, the intrinsic thermal conductivity of epoxy resins is usually lower than 0.2 Wm^−1^K^−1^ [6,7]. Under the application conditions of high temperature and high heat flow density, heat accumulation and thermal damage are easily generated [8,9], which seriously affects the safe and stable operation of the devices [10]. In addition, the thermal stability and abrasion resistance of epoxy resins under high-temperature environments is also very insufficient [11], which limits their further application in high-temperature structural components and thermal protection materials [12]. Therefore, enhancing the thermal management capability and thermal stability of epoxy resin materials has become an important research direction in the field of materials [13]. In order to improve the relevant properties of epoxy resins, researchers have commonly adopted the incorporation of different thermally conductive fillers into the resin system, which is placed between the chip and the heat sink in order to disperse these thermal energies [14]. However, both the type of filler and the distribution of the filler in the epoxy resin directly affect the formation of thermally conductive channels within the composite [15,16]. In this paper, graphene [17,18,19] and boron nitride [20] with high thermal conductivity and thermal stability were selected as fillers while reducing the disordered dispersion of fillers in the epoxy resin and the thermal resistance between fillers [21]. In this paper, graphene/boron nitride/iron oxide composite fillers with magnetically responsive properties were prepared by in situ growth of iron oxide nanoparticles on the surfaces of graphene and hexagonal boron nitride via liquid-phase co-precipitation method. The magneto-responsive fillers can be oriented along the direction of magnetic lines of force in the epoxy resin under the action of the external magnetic field and construct more dense and continuous thermal conductivity channels in the epoxy resin [22]. The thermal interfacial impedance between fillers is effectively reduced, and the overall thermal conductivity of the composites is improved. At the same time, graphene boron nitride in the filling of thermally conductive filler in the composite material can quickly disperse heat when the local temperature rises, avoiding the formation of local high-temperature points, thus reducing the decomposition due to the accumulation of heat [23]; high temperature can also form a layer of physical barriers to prevent the further diffusion of oxygen, heat to the interior of the epoxy matrix, inhibit the thermo-oxidative degradation reaction, and enhance the material’s resistance to ablation performance [24].

In this paper, by enhancing the distribution arrangement orderliness of magnetically responsive fillers in epoxy resin, the thermal conductivity is enhanced by more than nine times compared with pure epoxy resin. The thermal conductivity and thermal stability of epoxy resin are effectively improved, which provides a material basis for the high performance of epoxy resin in the field of electronic device encapsulation and high-temperature protection and also provides a theoretical basis and practical reference for expanding the application of magnetic field-assisted functional fillers in polymer composites.

## 2. Materials and Methods

### 2.1. Experimental Materials

Epoxy resin with a viscosity of 6000 CPS at 25 °C and a curing agent with a viscosity of 200 CPS at 25 °C were purchased from Shandong Yusuo Chemical Technology Co., Dezhou, China; graphene (about 3–4 layers, average 9.65 per micron) was procured from Guangxi TsingLube New Material Technology Co., Ltd., Liuzhou, China; boron nitride (2–3 micron) was procured from Shanghai Dinago Alloy Material Co., Ltd., Shanghai, China; Polystyrene Sulfonic Acid was procured from Anhui Kool Biological Engineering Co., Ltd., Anqing, China; ferrous sulfate heptahydrate, sodium hydroxide, and anhydrous ethanol were purchased from Xilong Chemical Co., Shantou, China; Hydrochloric acid was purchased from Shanghai McLean Biochemical Technology Co., Shanghai, China.

### 2.2. Preparation and Characterisation of Magnetically Responsive Fillers

#### 2.2.1. Preparation of Magnetically Responsive Fillers

A total of 10 g of the graphene/boron nitride filler prepared above was dissolved in 200 mL of aqueous ethanol solution, in which the volume ratio of anhydrous ethanol to ultrapure water was 1:1, and ultrasonically dispersed. A total of 10 g of ferric chloride hexahydrate was taken with 5 g of ferrous sulfide heptahydrate, dissolved in 50 mL of ultrapure water, placed on a magnetic stirrer, and stirred thoroughly. The graphene/boron nitride filler treated with sodium polyvinylbenzene sulfonate was mixed with ferric chloride hexahydrate and ferrous chloride hexahydrate solution and poured into a flat-bottom flask, sealed and isolated to prevent oxygen from oxidising divalent ferric ions to trivalent ferric ions, and stirred for 2 h. A total of 5 g of sodium hydroxide was dissolved in 50 mL of ultrapure water, the PH of the compound solution with a PH agent was measured, and the aqueous sodium hydroxide solution was added slowly to adjust the PH of the compound solution to about 13. Stirring was continued slowly for 15 min, and the bottom flask was placed in a vacuum-drying oven with the temperature adjusted to 60–70 °C to keep warm for a few hours until the solids in the flask were completely precipitated, at which time the following reaction occurred on the graphene/boron nitride surface:

Fe^2+^ + 2Fe^3+^ + 8OH^−^ − Fe_3_O_4_ + 4H_2_O (1)

**Figure 1:**
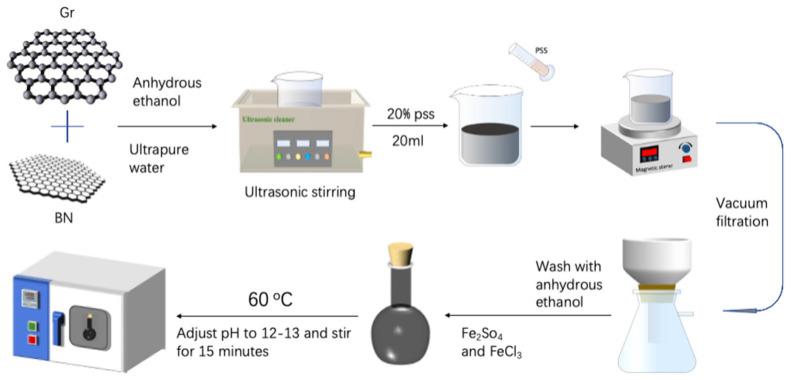
The preparation process of the magnetic response filler.

The solid–liquid mixture at the end of the reaction, the solid obtained by vacuum filtration, was washed several times and dried to obtain M-G-BN filler after measuring the pH neutral, as shown in Figure 1:

#### 2.2.2. Preparation of Magnetically Responsive Filler Composite Epoxy Materials

**Figure 2:**
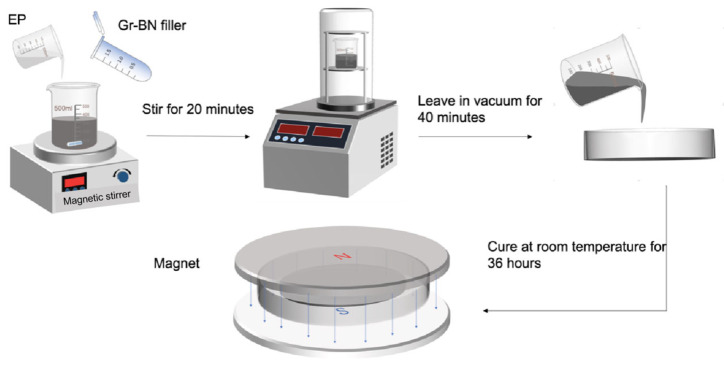
Preparation of magnetic responsive filler composite epoxy material.

**Figure 3:**
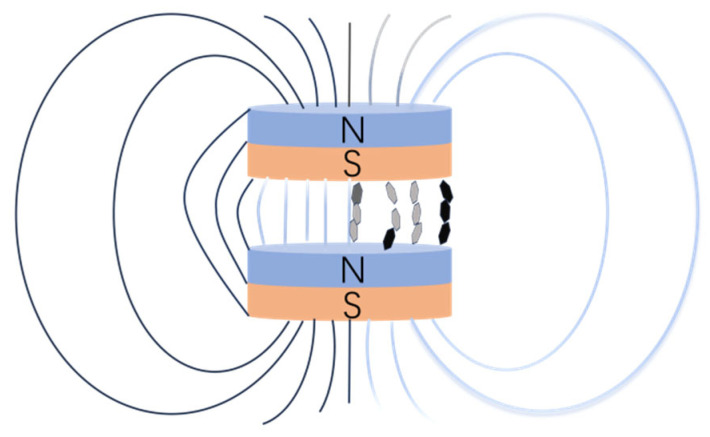
Schematic diagram of magnetic field-assisted curing.

As shown in Figure 2, it is the process of preparing epoxy resin composite materials. Figure 3 shows the distribution of fillers in epoxy resin during magnetic field-assisted curing. The epoxy resin and the epoxy resin curing agent were weighed in accordance with the weight ratio 3:1, and the mixture was followed by the addition of an appropriate amount of magnetically responsive graphene/boron nitride/iron oxide composite filler, such that the filler accounted for a mass percentage of the resin system of 0%, 5%, 10%, 15%, 20%, 25%, and 30%, respectively. The filler and epoxy resin mixture was then mechanically stirred for 20 min and then placed in a vacuum drying oven for 40 min to reduce the air bubbles inside the epoxy resin. Finally, appropriate amounts of M-G-BN-epoxy resin mixtures with different percentages of fillers were poured into circular moulds, where the diameter of the moulds was 50 mm. The mould was then placed between magnets and cured with the aid of a magnetic field at room temperature for 36 h to prepare the epoxy resin composite. Consistent with the above preparation steps, as a comparison group test, the epoxy resin was weighed in accordance with the weight ratio 3:1 with the epoxy resin curing agent, and the mixture was followed by the addition of an appropriate amount of pure cubic boron nitride filler. The mass percentage of the filler in the resin system was made to be 0%, 5%, 10%, 15%, 20%, 25%, and 30%, respectively. The filler and epoxy resin mixture was then mechanically stirred for 20 min and then placed in a vacuum drying oven to evacuate for 40 min to reduce the air bubbles inside the epoxy resin [25], after which it was poured into a circular mould for curing at room temperature for 24 h. Magnetically responsive graphene boron nitride epoxy composites with 0, 5, 10, 15, 20, and 25% filler mass ratios were also prepared, but without utilising magnetic field-assisted curing.

### 2.3. Characterisation and Testing of Materials

#### 2.3.1. Characterisation of Fillers

The morphology of the magnetically responsive filler and the elemental composition of the filler were analysed using a scanning electron microscope/energy spectrometer, SIGMA 300, Carl Zeiss, Wetzlar, Germany; the filler was characterised by X-ray diffraction D8 Advance, Bruker, Berlin, Germany and the test conditions were set to a scanning angle of 5 degrees to 90 degrees, a scanning speed of 8 degrees per minute, and a loading voltage of 40 KV; the composition of the filler was analysed by Raman, XploRAplus, Horibafrance, Palaiseau, France; and the filler was analysed by XPS, Thermo Scientific K-Alpha, Waltham, MA, USA, for the presence of iron tetraoxide. The saturation magnetisation intensity of the filler was measured using a vibrating sample magnetometer, Model 8600, Lake Shore Cryotronics, Westerville, OH, USA.

#### 2.3.2. Performance Testing of Epoxy Composites

The thermal conductivity of different composites was tested using the DRP-2 thermal conductivity tester, Model DRP-2, Xiangtan Instrumentation Co., Ltd., Xiangtan, China, in which the test samples had a diameter of 50 mm and a thickness of between 4 and 5 mm. The thermal stability of the composites was tested using a simultaneous thermal analyser, NETZSCH STA449, NETZSCH Scientific Instruments Co., Ltd., Waldkraiburg, Germany, and the surface ablation morphology of the composites was characterised using a research-grade orthogonal materials microscope, ZEISS Axioscope 5, Carl Zeiss, Wetzlar, Germany, and a white light interferometer, BRUKER, Berlin, Germany.

## 3. Results and Discussion

### 3.1. Characterisation of Magnetically Responsive Packings

**Figure 4:**
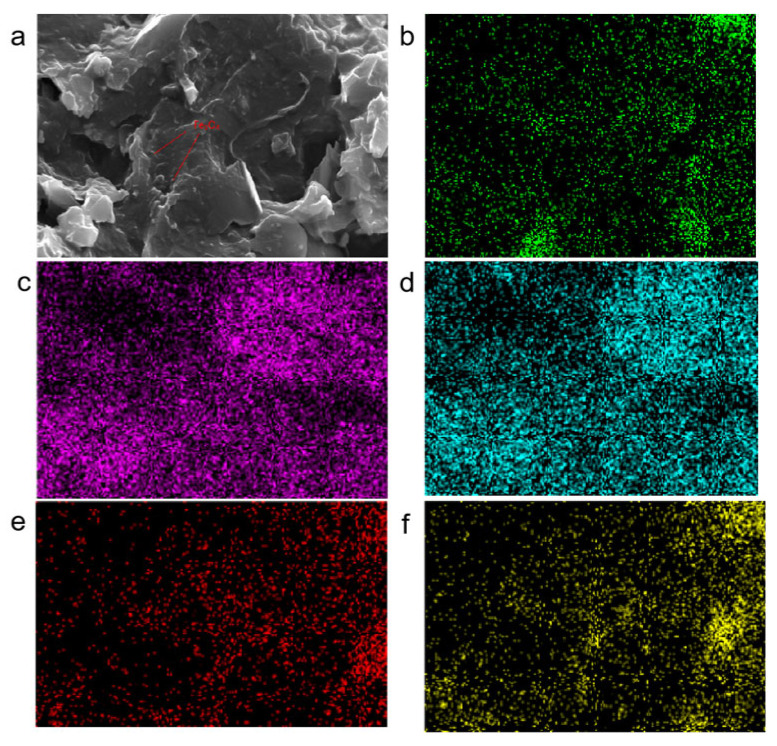
EDS characterisation of magnetically responsive graphene/boron nitride/iron oxide fillers. (**a**) Scanning electron microscope image of the packing material; (**b**) Carbon element in the composite packing material; (**c**) Nitrogen element in the composite packing material; (**d**) Boron element in the composite packing material; (**e**) Iron element contained on the surface of the composite packing material; (**f**) Oxygen element on the surface of the composite packing material.

An appropriate amount of prepared magnetically responsive graphene/boron nitride/iron oxide composite filler was taken, and the elemental composition contained in the prepared filler was analysed using EDS characterisation. As shown in Figure 4a below in the scanning electron micrograph (SEM) of the filler, it can be seen that there are many fine raised particles distributed on the surface of the composite filler, which is different from the two-dimensional planar structure of graphene and boron nitride. The possible reason for their formation was analysed to be the substances generated by the chemical reaction between trivalent and divalent iron ions on the surface of graphene and boron nitride in an alkaline environment, which gradually aggregated into visible iron oxides. Further elements that may be present on the surface of magnetically responsive graphene/boron nitride/iron oxide composite filler were elementally analysed, as shown in the following Figure 4b–f EDS elemental analysis. Where Figure 4b green represents the carbon element in the composite filler, Figure 4c pink is the nitrogen element in the composite filler, Figure 4d represents the boron element in the composite filler, Figure 4e represents the iron element contained on the surface of the composite filler, and Figure 4f represents the oxygen element on the surface of the composite filler. According to the distribution of colours on the EDS spectrum, the carbon, nitrogen, boron, iron, and oxygen elements on the surface of the composite filler are more evenly distributed. Because the composite filler is mainly composed of graphene and boron nitride, the filler contains a large number of carbon, nitrogen, and boron elements. Composite filler surface contains a large number of iron and oxygen elements. It can be initially judged that the surface of the composite filler in the preparation process, trivalent iron ions, and divalent iron ions in the alkaline regulation of the chemical reaction between the formation of iron oxides.

**Figure 5:**
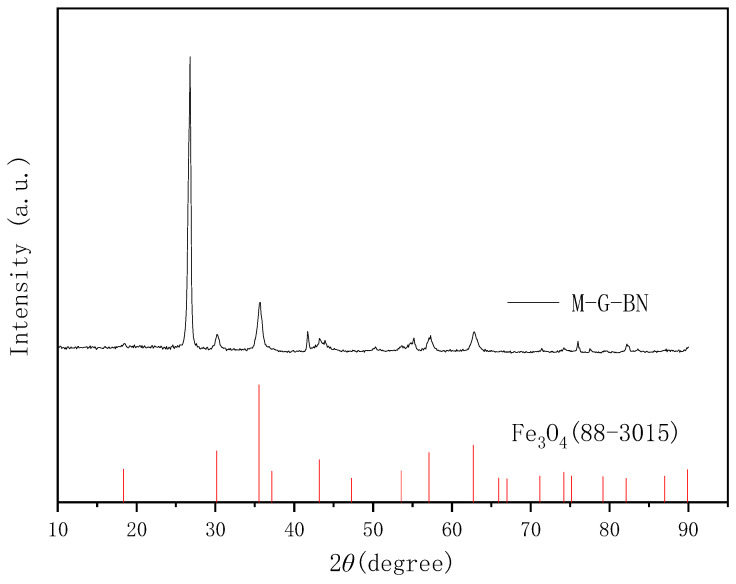
XRD characterisation of fillers.

Due to the fact that many iron oxides can be formed between iron and oxygen, it was not possible to determine which iron oxides were grown on the graphene and boron nitride surfaces by EDS elemental analysis alone. Therefore further XRD scanning analysis of magnetically responsive graphene/boron nitride/iron oxide filler, as well as Raman spectroscopy, were carried out to obtain the exact composition of the possible iron oxides. The results are shown in Figure 5, and a comparison with the standard PDF card shows that the magnetic responsive filler can be determined to have the characteristic peaks of iron oxide.

**Figure 6:**
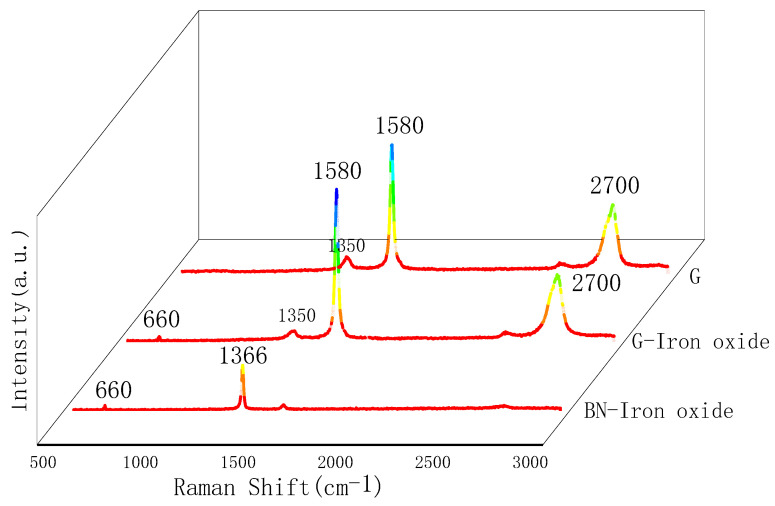
Raman characterisation of fillers.

Figure 6 shows the Raman spectrum of the magnetically responsive filler. The results indicate the presence of magnetite (Fe_3_O_4_) within the magnetically responsive graphene/boron nitride/iron oxide fillers. To further elucidate the surface composition of the graphene/boron nitride composite fillers, Raman spectroscopy was conducted, and the results are presented in Figure 6.

The Raman spectrum reveals characteristic peaks corresponding to graphene at approximately 1350 cm^−1^ (D band), 1580 cm^−1^ (G band), and 2700 cm^−1^ (2D band), while hexagonal boron nitride exhibits a peak near 1366 cm^−1^. Additionally, a prominent peak observed around 660 cm^−1^ suggests the presence of iron oxides on the filler surface, which may be attributed to FeO or Fe_3_O_4_ [26]. Moreover, the intensity ratio of the D band to the G band (ID/IG) for graphene in the filler is approximately 0.09, which is significantly less than 1. This indicates that the structure of graphene remains largely intact during the alkaline synthesis process of the magnetically responsive G-BN-Fe fillers, implying minimal structural damage to the graphene framework.

**Figure 7:**
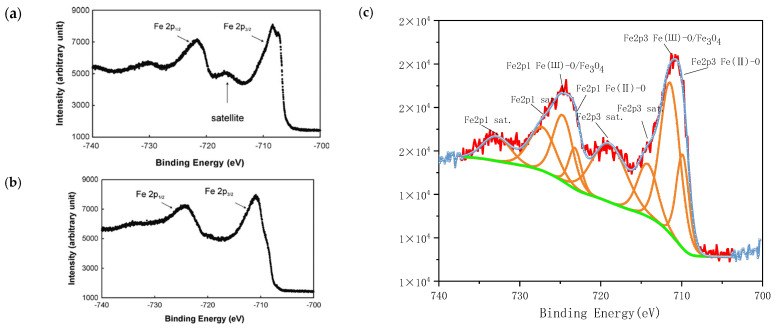

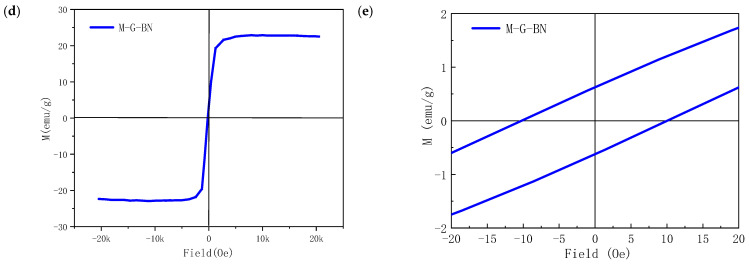
XPS analysis and magnetisation curve of magnetic response packing. (**a**) The XPS energy spectrum of standard ferric oxide [27]. (**b**) The standard XPS spectrum of magnetite [27]. (**c**) XPS patterns of fillers. (**d**,**e**) Magnetisation curve.

As shown in Figure 7c, a comparison between the XPS spectrum of the prepared magnetically responsive filler and the standard spectra of ferric oxide (Fe_2_O_3_) and magnetite (Fe_3_O_4_) reveals distinct spectral features characteristic of Fe_3_O_4_, indicating the presence of magnetite in the sample. Previous studies have reported that Fe_3_O_4_ typically does not exhibit satellite peaks at the Fe 2p_3_/_2_ binding energy. However, in the present study, multiple satellite peaks are observed, which may be attributed to the coexistence of other iron oxide phases, such as Fe_2_O_3_, thereby introducing spectral interference. Specifically, the XPS spectrum shows a Fe(II)-O characteristic peak at 709.88 eV corresponding to Fe 2p_3_/_2_, indicating the presence of Fe^2+^ species. A prominent peak at 711.42 eV is assigned to Fe(III)-O/Fe_3_O_4_, confirming the existence of Fe^3+^. Additionally, peaks at 723.19 eV and 724.69 eV correspond to Fe(III)-O and Fe(III)-O/Fe_3_O_4_ in the Fe2p_1_ region, respectively. Satellite peaks are also observed at 714.22 eV and 718.96 eV (Fe2p_3_), as well as at approximately 727.21 eV and 732.85 eV (Fe2p_1_). These spectral features collectively demonstrate that the synthesised magnetically responsive filler is primarily composed of Fe_3_O_4_, with a minor presence of Fe_2_O_3_, which accounts for the observed satellite peaks.

The magnetisation curve of the packing is shown in Figure 7d, and the saturation magnetisation intensity of the packing is approximately 22 emu/g. Meanwhile, as shown in Figure 7e, it can be understood that the coercive force of the magnetic response filler is relatively small, indicating that the prepared material belongs to the category of soft magnetic materials, and the material is easy to magnetise and demagnetise.

Based on the results presented, we successfully achieved the growth of iron oxide on the surfaces of graphene and boron nitride under liquid-phase conditions. Through characterisation and analysis using scanning electron microscopy (SEM), energy dispersive spectroscopy (EDS), X-ray diffraction (XRD), Raman spectroscopy, and X-ray photoelectron spectroscopy (XPS), we have gained insights into the ability of graphene and boron nitride surfaces to support the growth of the magnetically responsive material, iron oxide.

### 3.2. Thermal Conductivity Test

**Figure 8:**
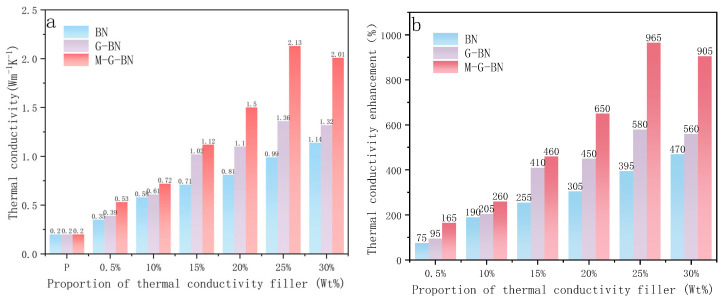
The thermal conductivity of composites with different filler ratios and the percentage improvement of thermal conductivity. (**a**) Thermal conductivity graph. (**b**) Percentage improvement of intrinsic thermal conductivity relative to pure epoxy resin.

As shown in Figure 8, BN indicates that the added thermally conductive filler is boron nitride, G-BN indicates that no magnetic field-assisted curing is utilised, and M-G-BN indicates that magnetic field-assisted curing is utilised. As a comparison group, the thermal conductivity of the pure epoxy resin can be measured to be relatively low at about 0.2 Wm^−1^K^−1^. When hexagonal boron nitride was used as a thermally conductive filler, the thermal conductivity of the composite was measured to be about 0.35 Wm^−1^ K^−1^ when the mass ratio of the filler was 5%. Meanwhile, as shown in Figure 8a, when the content of hexagonal boron nitride is increased to 10%, the thermal conductivity of the composite material increases to 0.58 Wm^−1^K^−1^, and with the content of hexagonal boron nitride gradually increases from 10 to 25%, the thermal conductivity of the composite material is also gradually increased, from 0.71 Wm^−1^K^−1^ to 0.81 Wm^−1^K^−1^, 0.99 Wm^−1^K^−1^ and 1.44 Wm^−1^K^−1^. The experimental results show that the thermal conductivity of the filled thermally conductive polymer composites is positively correlated with their filler content, and at the same time, there exists a certain threshold value of filler content below which the thermal conductivity of the composites is not significantly improved. At the same time, we can find that with the further enhancement of the boron nitride content, the thermal conductivity of the composites is not significantly improved as expected. When the thermally conductive filler is replaced with the prepared magnetically responsive filler, the thermal conductivity of the composite is about 0.39 Wm^−1^K^−1^ when the mass ratio of the filler is 5%. The increase in thermal conductivity compared to the boron nitride filler may be attributed to the high intrinsic thermal conductivity of graphene, which forms a thermal conductive channel with boron nitride for faster heat transfer.

The thermal conductivity of the composites is increased to 0.5 Wm^−1^K^−1^ when the percentage of magnetically responsive filler is increased to 10%. Consistent with the change in thermal conductivity when boron nitride is used as a thermally conductive filler, the thermal conductivity increases as the filler mass percentage in the composite continues to increase. This is due to the fact that when the filler content increases, the fillers randomly contact each other at more contact locations, which in turn makes the formation of thermally conductive channels inside the composite easier. However, when the filler percentage is too high, the thermal conductivity decreases slightly. This may be due to the high percentage of thermally conductive fillers in the filler, resulting in agglomeration between the fillers due to the high viscosity of the epoxy resin composite system, as well as more prone to air bubbles inside the resin, increasing the thermal resistance of the material. The thermal conductivity of the composites can be further improved by using magnetically responsive fillers as the thermal conductive channels inside the composites and by adding curing agents and curing with the aid of a magnetic field. The thermal conductivity of the composites without magnetic field curing was about 0.39 Wm^−1^K^−1^ compared to that of the composites without magnetic field curing at a filler mass ratio of 5%, and the thermal conductivity was further improved to 0.53 W m^−1^K^−1^ with the addition of magnetic field. The thermal conductivity of the composites with 10, 15, 20, 25, and 30% filler ratios is also found to be higher than that of the composites without magnetic field-assisted curing for each group. At the same time, it can be found that when the filler ratio is around 25%, the thermal conductivity of the composite material is increased by as much as 965%, which is a significant advantage over the thermal conductivity increase after applying magnetic field-assisted curing compared to curing without magnetic field-assisted curing. However, similarly, when the filler percentage is around 30%, the percentage of thermal conductivity improvement decreases slightly. This is due to the fact that the external magnetic field will make the thermally conductive filler inside the epoxy resin in closer contact, and the orientation of the filler will be improved. The thermally conductive channel inside the composite material will provide a faster propagation path for phonon transmission, and the phonon scattering will be greatly reduced. However, for the same filler ratio, the thermal conductivity of the composites is significantly higher than that of the composites cured without an external magnetic field when a certain amount of magnetically responsive filler is added to the epoxy resin and cured with the aid of the magnetic field.

### 3.3. Mechanism of Magnetic Response Fillers to Enhance Thermal Conductivity of Epoxy Resin

**Figure 9:**
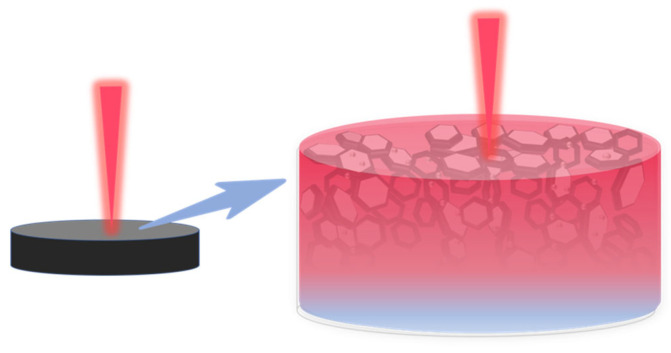
The mechanism of composite filler to enhance the thermal conductivity of epoxy resin.

As shown in Figure 9, during the magnetic field-assisted curing process, the magnetic response fillers inside the epoxy resin can contact each other more fully and closely, and the constructed heat conduction channels are more effective. When the external heat source comes into contact with the composite material, heat can be transferred more quickly in the epoxy resin composite material. It has been demonstrated by the above experiments that the use of prepared thermally conductive fillers as additives for improving the thermal conductivity of epoxy resins can enhance the thermal conductivity of composites. And it is shown by the experimental results that the mass percentage of graphene boron nitride filler inside the epoxy resin is improved. The thermal conductivity of the composites prepared without the curing stage using external magnetic field-assisted curing still has the effect of improving the thermal conductivity of the epoxy resin.

**Figure 10:**
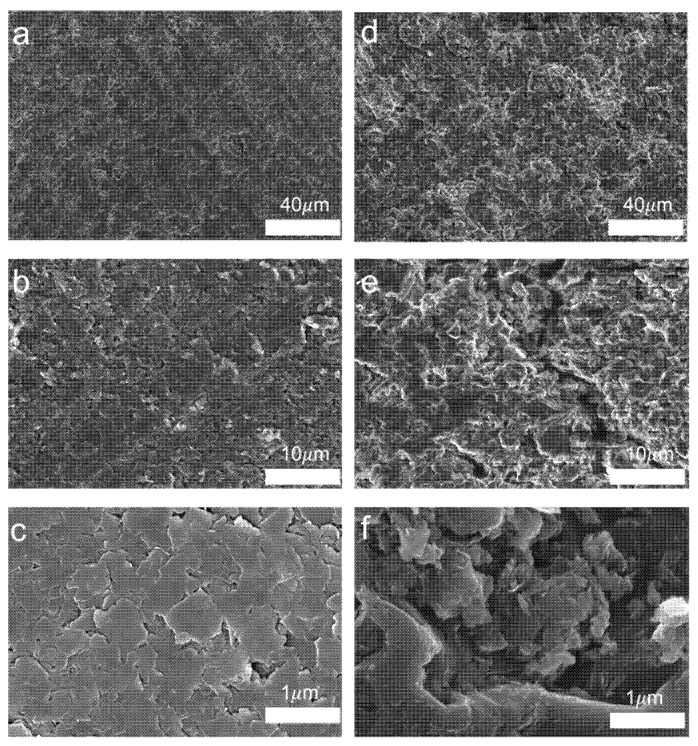
Distribution of fillers for magnetic field-assisted curing vs. no magnetic field-assisted curing: (**a**–**c**) 25% filler with magnetic field-assisted curing; (**d**–**f**) 25% filler without magnetic field-assisted curing.

As shown in Figure 10, when the heat source contacts the thermally conductive filler, a thermally conductive network is formed due to the filler inside the epoxy resin composite contacting each other. As the filler for epoxy resin composite thermal conductivity affects the filler in the epoxy resin distribution, the arrangement of the state has an important relationship, and the composite cross-section can be a direct response to the distribution of filler in the epoxy resin.

In this paper, scanning electron microscope scans are used to analyse the cross-sections of epoxy composites with a mass fraction of 25%. The mechanistic aspects are illustrated by comparing the difference in filler distribution between sections cured with and without magnetic field-assisted curing. Firstly, the cross-section of the composite was sprayed with a layer of metal, and then the surface was observed using SEM, and the cross-section of the composite was illustrated. As shown in Figure 10a below, when a magnetic field is applied, it can be observed that the filler section surface inside the epoxy resin is relatively flat, the contact between fillers is relatively tight, and an obvious stacking structure can be seen. This is probably because the graphene/boron nitride fillers in the flake structure under the action of the magnetic field can be arranged along the direction of the magnetic field and contact each other, which is conducive to the formation of a thermally conductive flux inside the epoxy resin.

As shown in Figure 10d above without using magnetic field-assisted curing, the composite cross-section fillers can be seen to be not so orderly and unevenly distributed. The reason for this is the lack of force orientation between the fillers before curing, which makes the random stacking of graphene and boron nitride in the lamellar structure with each other more prone to agglomeration effects. When further observing Figure 10b above, it can be found that the gap between the fillers is smaller under the effect of magnetic field-assisted curing, and the flake-like graphene nitride fillers are able to contact effectively. This can prevent the thermal conductive channels from being disconnected due to defects to enhance the thermal resistance. In contrast, in Figure 10e, due to the obvious gaps between the fillers, this can introduce defects inside the polymer that are unfavourable for phonon transmission. As in Figure 10c, the fillers inside the epoxy resin are closely aligned with each other. As shown in Figure 10f, the fillers may be stacked in random contact inside the resin due to uneven size, resulting in large gaps between the fillers. And the fillers randomly contact each other in the form of contact may be point-face contact, point contact that will make the contact thermal resistance become very large. Not conducive to the construction of the thermal conductivity network and thus cannot give full play to the filler’s thermal conductivity.

### 3.4. Thermal Stability of Magnetic Responsive Filler Composite Epoxy Resin Materials

**Figure 11:**
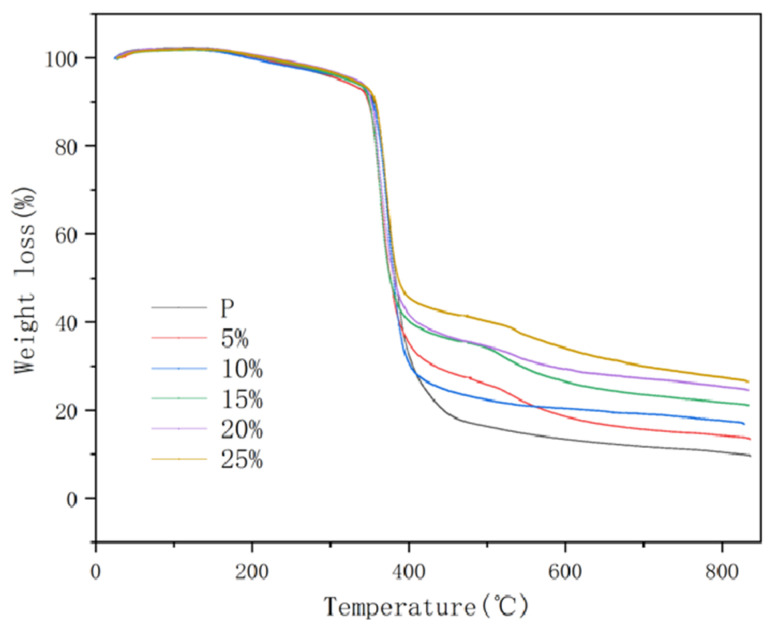
The thermogravimetric analysis diagram.

The thermogravimetric analysis for filler mass fractions of 0, 5, 10, 15, 20, and 25% is shown in Figure 11 below, in which the mass of the test samples of different test groups is taken as about 9.20 mg, the composites are put into the thermogravimetric analyser at an initial temperature of about 30 °C, the rate of temperature rise is 20 °C per minute, and the temperature naturally cools down after it rises to 800 °C.

The results show that the composites exhibit a single degradation process after the addition of graphene/boron nitride/iron oxide filler, which is the same as that of the pure epoxy resin, as can be seen from the figure. It shows that the introduced filler did not change the pyrolysis mechanism of the epoxy resin. The addition of thermally conductive fillers does not adversely affect the rate of thermal decomposition of epoxy resins compared to the decomposition of pure epoxy resins. The results show that the thermogravimetric curve is a straight line with increasing temperature in the range of 25 to 170 °C, and there is almost no mass change in the epoxy resin and the composites with different filler ratios, which suggests that there is almost no thermal decomposition of the composites. When the temperature is in the range of 170 and 350 °C, the thermogravimetric analysis curve of the pure epoxy resin and the filler group with different mass ratios increases slowly and can be approximated as a straight line. The hydrophilic groups (e.g., hydroxyl, carbonyl) in the epoxy resin material begin to decompose. The epoxy resin inside the composite material decomposes slowly, as in the case of thermally conductive fillers, with the maximum decomposition rate being reached at 350 °C. The decomposition rate of the pure epoxy resin slows down in the range of 350 to 450 °C. The decomposition rate becomes very slow, from 450 to 800 °C. The decomposition rate of the pure epoxy resin is much slower than the pure epoxy resin in the range of 450 to 800 °C. The decomposition rate of the pure epoxy resin in the range of 350 to 450 °C is much slower. Compared with the pure epoxy resin, the decomposition rate of the composite with thermally conductive fillers is much lower than that of the pure epoxy resin at 450 °C. At the same time, the composite material enters into a stage of rapid thermal decomposition from 350 to 400 °C. The decomposition rate of the composite material is much lower than that of the pure epoxy resin at 350 °C. At about 400 °C, it can be observed that the decomposition rate of the components with thermally conductive fillers starts to slow down and gradually tends to level off. Graphene and boron nitride are not easy to decompose or burn and can maintain structural integrity at high temperatures, which can help inhibit the spread of thermal decomposition of epoxy matrix and improve the thermal stability of epoxy resin to a certain extent. The close arrangement of graphene and boron nitride also prevents heat and oxygen from penetrating the interior, forming a “heat insulation layer” or “oxygen barrier”.

### 3.5. Ablation Resistance of Magnetic Responsive Filler Composite Epoxy Resin Materials Performance

#### 3.5.1. Ablation Morphology of Magnetically Responsive Filler Composite Epoxy Resin Materials

Pure epoxy resin and graphene/boron nitride/iron oxide epoxy resin composites with different mass ratios were placed at a distance of 2.5 cm from the outer flame of the windproof flame, and the flame was turned on to ablate the samples for 5 s, then the flame was turned off and the flame was extinguished on the surface of the materials. By comparing the ablation morphology and colour change of the surface of the pure epoxy resin material and the composite material with added thermally conductive filler, the difference in the ablation resistance of the materials was determined.

**Figure 12:**
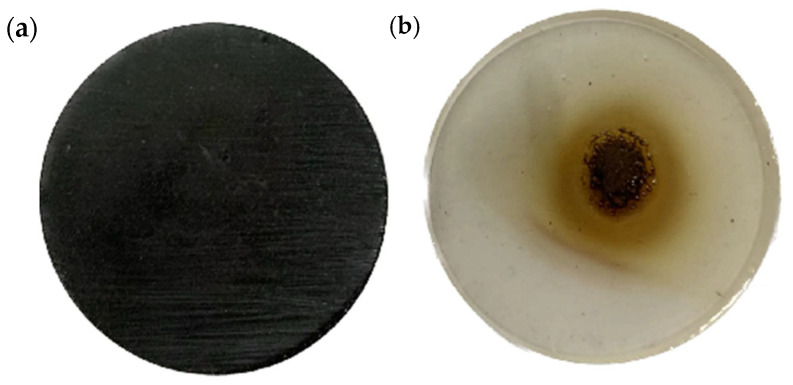
Ablation of composite material under magnetic field-assisted curing with 25% filler ratio vs. pure epoxy resin material ablation map. (**a**) Ablation of composites under magnetic field-assisted curing with 25% filler (**b**) Ablation of pure epoxy resin.

As shown in Figure 12a,b, the shape of the composite material changed significantly after the pure epoxy resin material was ablated under the flame, and there was a large amount of carbonised material on the surface formed after the epoxy resin was ablated. After adding thermally conductive filler into the epoxy resin material and then after flame ablation, the composite material still has different degrees of ablation, but compared with the pure epoxy resin, the ablation traces are much less. Pure epoxy resin has obvious ablation marks after 5 s of ablation under the windproof flame, and the ablation colour gradually turns yellow from black in the centre to the outside. The surface of the composite with a mass fraction of 25% has only a few ablation marks but no obvious uneven bubble holes, indicating that the ablation of the material is much improved compared to the situation with the pure epoxy resin. In order to further compare the ablation resistance of the epoxy resin material with a 25% mass ratio of magnetically responsive graphene/boron nitride/iron oxide filler with that of the pure epoxy resin. Next, the surface morphology of the ablation centre region of the pure epoxy resin and that of the epoxy resin composite with a 25% filler ratio were observed by optical microscopy, and the three-dimensional morphology of the ablated surfaces was characterised using white light interference.

**Figure 13:**
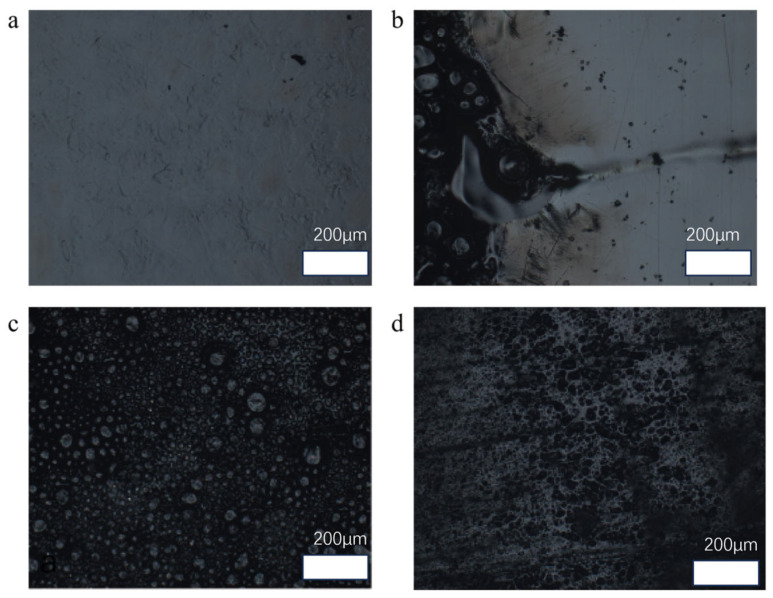
The ablative comparison of different materials. (**a**) The surface morphology of pure epoxy resin. (**b**,**c**) The ablation morphology of pure epoxy resin. (**d**) The ablation morphology of composite materials.

As shown in Figure 13a above, it can be observed under the optical microscope that the surface of pure epoxy resin before ablation is relatively flat and smooth, and the colour is transparent with a uniform overall colour. After ablation, as shown in Figure 13b, it can be observed that the colour change at the edge of the ablated area is obvious, showing gradual yellow and uneven, which can be reflected according to the change in colour about the degree of ablation near the centre is more serious. Moreover, the centre area of the epoxy resin is no longer flat and smooth after ablation, but the surface contains a large number of pits and bumps in the shape of bubbles. Meanwhile, under the optical microscope, as shown in Figure 13c, the surface of the pure epoxy resin before ablation is very different from the flat and smooth surface, and the surface of the pure epoxy resin after ablation has obviously raised bubbles. This is because the epoxy resin itself is made of large molecules containing epoxy group polymerisation, containing a large number of functional groups and small molecules, internal high-temperature combustion when the epoxy resin will easily produce many kinds of gases inside [28]. When the gases generated inside the epoxy resin at high temperatures expand outward, the outer layer of the resin softens at high temperatures and prevents the release of the gases outward, which results in the creation of bubbles and voids on the surface of the resin [29]. This also indicates that the epoxy resin itself has poor resistance to high-temperature ablation. Using an optical microscope to simultaneously observe the composite filler mass ratio of 25% of the epoxy resin material after ablation surface morphology, as shown in Figure 13d, when the addition of heat-conducting filler epoxy resin after the centre of the high-temperature ablation, although it is impossible to avoid the high temperature of the burned surface there is a certain amount of craters, but the surface of the composite material is not as uneven as after the ablation of the pure epoxy resin, but there are only a small number of There are only a few traces of ablation. Due to the addition of graphene/boron nitride/iron oxide filler, the epoxy composites are darker in colour, and therefore it is difficult to judge the ablation resistance based on the comparison of the colour change between the ablated and non-ablated areas. In order to further characterise the morphological changes in the ablation region, the central region of the contrast ablation surface was again observed using white light interference scanning.

**Figure 14:**
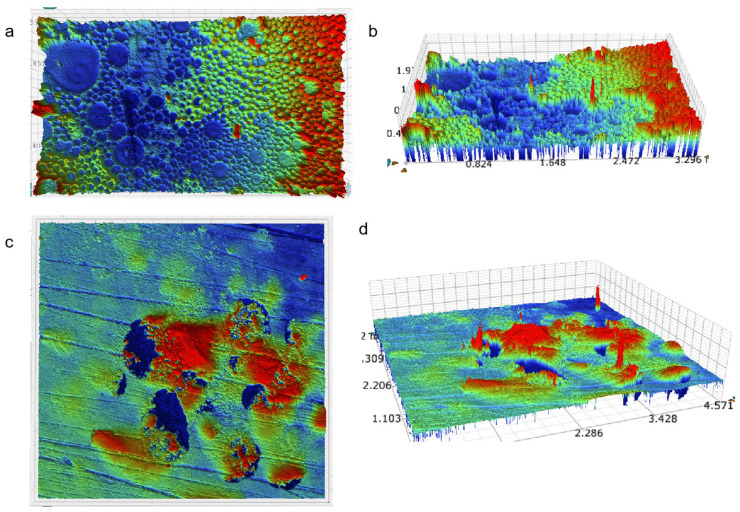
White light interference characterisation of surface ablation of pure epoxy resin with a 25% mass ratio of material. (**a**) P-EP; (**b**) P-EP; (**c**) 25; (**d**) 25%.

The three-dimensional morphology of the ablated surface of pure epoxy resin after a short period of high-temperature ablation can be more clearly observed under the characterisation of the white light interferometer. As shown in Figure 14a,b, the pure epoxy resin has a large number of obvious pits on the deeper surface in the centre of the ablation area. At the same time, as shown in Figure 14c,d by observing the three-dimensional stereo image, it can be clearly found that, different from the pure epoxy resin surface, there are a large number of pits left after ablation, add filler composite surface still exists in part of the pits left after ablation, but the relative ablation pits area is also less, the surface of the composite material to receive the impact of the scorching is small. It shows that the ablation resistance of the composite material is stronger than that of the pure epoxy resin material.

#### 3.5.2. Anti-Burnout Mechanism of Magnetic Response Filler Composite Epoxy Resin Materials

**Figure 15:**
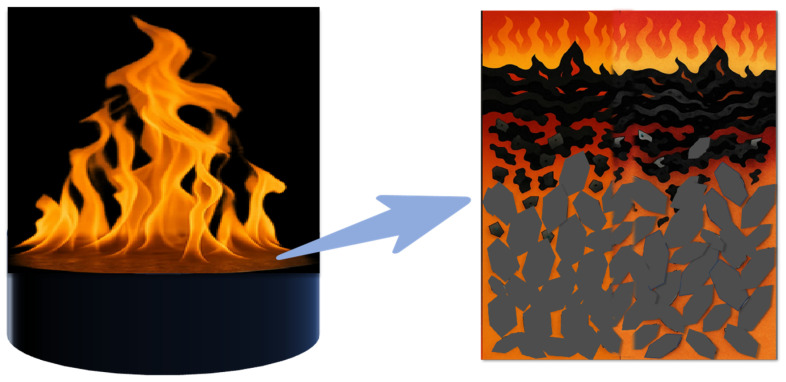
The anti-ablative mechanism of the packing.

The reason for the enhancement of the prepared graphene/boron nitride/iron oxide filler for the ablation resistance of the epoxy resin may be shown in the mechanism schematic in Figure 15. When an external flame burns the deposited epoxy resin, the pure epoxy resin lacks the protective shielding effect of the filler, resulting in its direct mutual contact with the external flame. In addition, the low thermal conductivity of the pure epoxy resin itself makes the pure epoxy resin material unable to cope with the surge of heat quickly, which leads to intense combustion of the epoxy resin and more serious ablation. Sang Hyuk Yum et al. [30] studied the ablation resistance of phenol-formaldehyde and epoxy-based polymer matrices by combining the two types of nanotubes (MWCNTs, BNNTs) and the two types of polymer resins (phenol, epoxy) were combined to prepare four different types of nanocomposites. It was found that MWCNTs significantly improved the ablative resistance of the epoxy matrix, allowing MWCNT/epoxy to obtain ablative resistance comparable to that of MWCNT/phenol nanocomposites. Meanwhile, the paper illustrates that the BNNT fillers have excellent thermal stability unmatched by MWCNT, which is the determining factor of the ablative resistance of the polymer matrix, as well as the distribution state of the nanotube fillers, is the main factor determining the higher ablative resistance of MWCNT/epoxy. It is shown that the thermal stability of the fillers in the polymer and the distribution of the fillers in the polymer have an effect on the corrosion resistance of the composites.

In the experiment, the inside of the epoxy resin was filled with graphene/boron nitride/iron oxide filler, and the graphene and boron nitride themselves have good thermal stability, especially the ceramic filler boron nitride. At the same time, in the magnetic field-assisted curing process, graphene/boron nitride/iron oxide fillers are arranged in an orderly manner, and the thermally conductive channels constructed by the thermally conductive fillers inside them reduce the overall temperature gradient of the material to a certain extent. Coupled with the high intrinsic thermal conductivity of graphene and boron nitride can quickly disperse the heat of the composite material in contact with the flame electrical position to the whole surface. The heat can be timely conducted apportioned to other internal materials, avoiding the thermal decomposition of epoxy resin caused by the high temperature of the local temperature as well as reducing the softening and burning of the resin due to the concentration of the temperature. Aspen N. Reyes [31] et al. use boron nitride carbon nanotubes of high strength and modulus as well as high thermal stability and oxidation temperature properties by preparing two hybrid composites with satin-woven carbon fibre (CF) and heavy phenolic resin: a surface layer of BNNT and an alternating interlayer of BNNT. The thermal diffusion efficiency was improved while the carbon nanotubes in the surface layer strengthened the carbon crystalline oxide structure and reduced the oxygen diffusion. In the experiment, because the outer graphene boron nitride has a high ignition point, graphene and boron nitride can play a role in isolating the flame to a certain extent, reducing the flame on the internal epoxy resin material ablation. Secondly, graphene and boron nitride in close contact with each other inside the epoxy resin can also be further blocked in the heat flow and oxygen intrusion. The carbon layer formed after combustion also has a high heat resistance and is well saturated with internal material combustion [32]. Therefore, the thermally conductive filler enhances the fast thermal response of the composite material and, at the same time, plays a good barrier shielding role to effectively enhance the ablation resistance of the epoxy resin.

## 4. Conclusions

In this paper, graphene/boron nitride/iron oxide composite fillers with magnetic response performance were prepared by liquid-phase co-precipitation method and introduced into the epoxy resin system. The ordered arrangement of the fillers in the resin was achieved with the help of an external magnetic field, thereby constructing an efficient heat conduction channel. Research shows that when the mass proportion of the filler is 25%, and magnetic field-assisted curing is adopted, the thermal conductivity of the composite material significantly increases to 2.13 Wm^−1^K^−1^, approximately 9.65 times that of pure epoxy resin while maintaining high electrical insulation. In addition, after the introduction of this composite filler, the thermal stability and ablation resistance of the material have also been significantly enhanced. This research provides new ideas and technical support for the design and application of polymer composites with high thermal conductivity, high insulation, and high-temperature resistance.

## Data Availability

Data will be made available on request.
